# Repetitive Blast Exposure Produces White Matter Axon Damage without Subsequent Myelin Remodeling: *In Vivo* Analysis of Brain Injury Using Fluorescent Reporter Mice

**DOI:** 10.1089/neur.2020.0058

**Published:** 2021-03-31

**Authors:** Donald V. Bradshaw, Yeonho Kim, Amanda Fu, Christina M. Marion, Kryslaine L. Radomski, Joseph T. McCabe, Regina C. Armstrong

**Affiliations:** ^1^ Graduate Program in Neuroscience, Physiology, and Genetics, Uniformed Services University of the Health Sciences, Bethesda, Maryland, USA.; ^2^ Center for Neuroscience and Regenerative Medicine, Physiology, and Genetics, Uniformed Services University of the Health Sciences, Bethesda, Maryland, USA.; ^3^ Department of Anatomy, Physiology, and Genetics, Uniformed Services University of the Health Sciences, Bethesda, Maryland, USA.; ^4^ Department of Neuroscience, Wexner Medical Center, Ohio State University, Columbus, Ohio, USA.

**Keywords:** axon damage, blast exposure, myelin, transgenic reporter mice, traumatic brain injury, white matter

## Abstract

The potential effects of blast exposure on the brain health of military personnel have raised concerns and led to increased surveillance of blast exposures. Neuroimaging studies have reported white matter abnormalities in brains of service members with a history of blast exposure. However, blast effects on white matter microstructure remain poorly understood. As a novel approach to screen for white matter effects, transgenic mice that express fluorescent reporters to sensitively detect axon damage and myelin remodeling were exposed to simulated repetitive blasts (once/day on 5 consecutive days). Axons were visualized using *Thy1-YFP-16* reporter mice that express yellow fluorescent protein (YFP) in a broad spectrum of neurons. Swelling along damaged axons forms varicosities that fill with YFP. The frequency and size of axonal varicosities were significantly increased in the corpus callosum (CC) and cingulum at 3 days after the final blast exposure, versus in sham procedures. CC immunolabeling for reactive astrocyte and microglial markers was also significantly increased. *NG2CreER;mTmG* mice were given tamoxifen (TMX) on days 2 and 3 after the final blast to induce fluorescent labeling of newly synthesized myelin membranes, indicating plasticity and/or repair. Myelin synthesis was not altered in the CC over the intervening 4 or 8 weeks after repetitive blast exposure. These experiments show the advantages of transgenic reporter mice for analysis of white matter injury that detects subtle, diffuse axon damage and the dynamic nature of myelin sheaths. These results show that repetitive low-level blast exposures produce infrequent but significant axon damage along with neuroinflammation in white matter.

## Introduction

Traumatic brain injury (TBI) is a significant public health concern for civilian and military populations. Brain injury associated with an explosive blast raises additional concerns due to the unknown effects of blast exposures and the potential for long-term symptoms.^1,2^ High explosives can result in injury from the blast wave along with result in injury to the head from projectiles striking the head or from the head hitting objects when the body is thrown. TBI associated with high explosive blasts accounted for approximately one-third of the nearly 400,000 military TBIs between 2001 and 2008.^3^ The effects of repetitive exposure to occupational blasts are an ongoing concern within the military. The National Defense Authorization Act (2018–2020) called for occupational surveillance to evaluate blast exposure, for example, among those engaged in breaching exercises or firing of heavy artillery.^4^ Early studies indicated a potential correlation between military personnel exposed to repeated low-level blast exposure and worsened long-term neurological and cognitive outcomes.^5,6^

The long-term effects of blast exposure on brain health remain poorly understood. Magnetic resonance imaging (MRI) studies using diffusion tensor imaging (DTI) detected changes in major white matter tracts of the brain in some deployed soldiers who experienced TBI with blast exposure.^7–9^ Studies of blast-exposed veterans with and without TBI found that blast exposure decreased DTI fractional anisotropy in several white matter tracts.^10^ Both breachers and veterans examined more than 1 year after blast exposure with TBI exhibited DTI changes in white matter.^11,12^ Further, veterans with blast-related mild TBI and loss of consciousness had increased white matter abnormalities and cognitive impairments.^13^

Post-mortem studies of brains of service members who experienced blast-related TBI have identified multiple neuropathological features. Axon damage, identified as axon swellings with accumulated β-amyloid precursor protein (β-APP), has been observed along with disruptions to the myelin that ensheaths axons.^14,15^ Tau, a protein that is bound to microtubules in the cytoskeleton of healthy axons, has been reported to become hyperphosphorylated and form aggregates in neurons in the brains of some veterans who experienced repetitive head injury and blast exposure.^16^ Additionally, brains from military service members with a history of blast exposure exhibited astroglial scarring at interfaces between tissues of differing densities; this astrogliosis involved the subpial glial plate, penetrating cortical blood vessels, structures lining the ventricles, and white matter–gray matter junctions.^17^ Together, these neuroimaging and neuropathology studies indicate vulnerability of the brain to blast exposure and the potential for axons and white matter to be critical pathological factors. However, the full range of neuropathological effects of repetitive blast exposure is challenging to determine because potentially subtle pathological features may be sparsely distributed throughout the brain, and may change over time. To overcome these challenges, we evaluated the effects of blast exposure on axon and white matter pathology using two lines of transgenic mice with genetic reporter constructs that enable detection of *in vivo* changes in specific cellular processes.

*Thy1-YFP-16* reporter mice express yellow fluorescent protein (YFP) in a broad spectrum of neurons so that individual axons can be visualized in diverse brain regions, including the corpus callosum (CC).^18–21^ We used *Thy1-YFP-16* reporter mice to screen for individual damaged axons across the brain and brainstem. YFP-filled enlargements form at sites of damage and result in swelling and formation of varicosities along axons. The localization of YFP in axon swellings is a sensitive indicator of axonal injury ranging from small swellings observed early, when damage may still be reversible, through to intensely fluorescent terminal end bulbs of disconnected axons.^22,23^ Our previous work in *Thy1-YFP-16* mice demonstrated YFP detection of traumatic axonal injury in white and gray matter regions of the brain at 3 days after single or repetitive concussive TBI.^20,21^ Importantly, in comparison with β-APP immunohistochemistry used for standard neuropathology, confocal analysis in *Thy1-YFP-16* mice can visualize YFP in three dimensions to identify terminal end bulbs at sites where axon damage has progressed to disconnection.^24^

Larger axons in white matter are wrapped by myelin sheaths that increase the efficiency and speed of action potential conduction. Myelin can be damaged by direct insults or as a secondary effect of axon damage. New myelin can be generated during activity-dependent adult plasticity and can form after injury or disease through regeneration and replacement of lost myelin sheaths.^25–29^ *NG2CreER;mTmG* transgenic fluorescent reporter mice enable *in vivo* analysis of newly formed myelin sheaths over a specific time interval.^21,30^ The *NG2* promoter is expressed in oligodendrocyte progenitor cells (OPCs) that proliferate and differentiate into myelin-forming oligodendrocytes. Tamoxifen (TMX) administration activates *Cre recombinase* that switches membrane fluorescent labeling from red to green in OPCs and their progeny. TMX given to *NG2CreER;mTmG* mice on days 2 and 3 after TBI results in green fluorescent protein (GFP) labeling of newly generated oligodendrocytes and their myelin sheaths.^21^ We previously showed that a single concussive TBI increased formation of new myelin in the CC through 4 weeks, yet CC atrophy was evident when survival was extended to 8 weeks after TBI.^21^ In this study we leveraged the advantages of the *NG2CreER;mTmG* mice to examine new myelin formation over 4 and 8 weeks after repetitive low-level blast exposure.

The effect of blast waves on the brains of *Thy1-YFP-16* and *NG2CreER;mTmG* mice was tested using an Advanced Blast Simulator (ABS) to model the blast wave component of low-level exposures to explosives or heavy artillery relative to potential effects of repetitive blast exposures during deployment or training. Studies of service members have shown that experiencing three or more TBI events, with or without blast exposure, may increase effects compared with those with fewer or no TBI history.^31,32^ In mice, our prior studies of concussive mild repetitive TBI, with impacts once per day on each of 5 consecutive days, resulted in persistent neuroinflammation in the CC and adjacent cerebral cortex.^20^ Therefore, the current studies in transgenic reporter lines used repetitive low-level blast exposures given once per day on each of 5 consecutive days for analysis of axon damage, myelination, and neuroinflammation.

## Methods

### Mice

All mice were treated in accordance with guidelines of the Uniformed Services University of the Health Sciences (USUHS) and the National Institutes of Health Guide for the Care and Use of Laboratory Animals. Mice were socially housed as 2–4 mice per cage in 35 cm × 16.5 cm × 18 cm cages that contained enrichment objects and maintained a 12-h daytime light cycle (0600–1800) during which all experimental procedures were performed. The following mouse strains were obtained from the Jackson Laboratory (Bar Harbor, ME, USA): *NG2CreER* (RRID: IMSR_JAX:008538; B6.Cg-Tg(Cspg4-cre/Esr1*)BAkik/J), *ROSA^mT/mG^* (RRID:IMSR_JAX:007676; B6.129(Cg)-*Gt(ROSA)26Sortm4(ACTB-tdTomato,-EGFP)Luo/J*), and *Thy1-YFP-16* (RRID:IMSR_JAX:003709; B6.Cg-Tg(Thy1-YFP)16Jrs/J). Mouse strains were bred as in-house colonies to generate experimental mice. First-generation offspring from *NG2CreER* and *ROSA^mTmG^* breeders were used as *NG2CreER;mTmG* mice. The total number of *Thy1-YFP-16* mice used was 15 (8 male, 7 female) and the total number of *NG2CreER;mTmG* mice used was 14 (10 male, 4 female). Male and female mice were combined for analyses after separate analyses showed no sex-based differences. Littermates of *Thy1-YFP-16* mice or *NG2CreER;mTmG* mice were used for quantitative analyses. The number of mice for each condition in each experiment is provided in each figure legend.

### Repetitive blast procedures

At 8–9 weeks of age, mice were randomly assigned to repetitive sham (rSham) or repetitive blast (rBlast) procedures. The rBlast mice were exposed to five consecutive simulated blast exposures, at 24-h intervals, delivered in an ABS (ORA, Inc., Fredericksburg, VA, USA). ABS procedures were performed with mice in the prone position for frontal blast exposure. Each mouse was anesthetized with 3% isoflurane and the head and body were secured to a wooden tongue depressor, wrapped in gauze, placed in mesh to prevent rotational movement, and positioned to face the oncoming blast as previously detailed.^33,34^ The rSham mice were anesthetized and the same procedures were followed except that the blast was not deployed. There was no significant difference in mean peak incident overpressure (21.7 ± 1.11 psi, ∼149.62 kPA), mean duration (7.25 ± 0.235 msec), or mean impulse (62.9 ± 0.795 psi-msec) across the five blast exposures for any groups. Righting reflex time was significantly prolonged in rBlast mice (mean = 78.55 sec) compared with rSham mice (mean = 45.8 sec) (two-way analysis of variance [ANOVA]; F(1,28) = 206.6; *p* > 0.0001). Righting reflex time did not change significantly across the 5 days of rBlast or rSham procedures for either genotype or injury condition.

### *Thy1-YFP-16* mice: Analysis of axon damage

At 3 days following the final rBlast or rSham procedure, *Thy1-YFP-16* mice were perfused with 4% paraformaldehyde and brains were cut as 14-μm-thick cryosections.^21^ For qualitative analysis of the distribution of axon damage, sagittal sections of *Thy1-YFP-16* mice were imaged using a Zeiss Axioscan Z.1 (Carl Zeiss) with a Plan-Apochromat 20 × objective to image the entire brain section, from olfactory bulb to brainstem. To examine the three-dimensional (3D) structure of YFP-filled axons, confocal image stacks were acquired on a 700 laser scanning confocal microscope (Carl Zeiss) with a Plan-Apochromat 40 × /1.4 oil or 63 × /1.4 oil objective and processed using Vision4D Modular Software (Arivis Vision4D, Phoenix, AZ, USA; RRID:SCR_018000).

Quantification of axonal varicosities focused on regions of interest (ROIs) in the CC and cingulum in coronal sections at levels between +0.5 and −0.5 mm relative to bregma. The CC ROI extended from the midline bilaterally to the turning point of ventral descent in the external capsule. The cingulum ROI was evident as fibers oriented perpendicular to the underlying CC and distinct from the superior cortical tissue. Images acquired on an Olympus IX-70 fluorescence microscope with a SPOT RT3 camera (Diagnostic Instruments, Arnold, MD, USA) were analyzed using Image J software (National Institutes of Health, Bethesda, MD, USA; RRID: SCR_003070). The ROI area was measured with a 10 × objective. Morphological measurements of YFP-filled swellings used images acquired with a 40 × objective. The width of each axonal varicosity was measured in the plane transverse to the length of the axon. The width of CC axons was <1.5 μm as measured by electron microscopy.^35,36^ Pilot analyses in rSham and rBlast mice confirmed that axon diameter varied along normal-appearing axons but ranged from 0.5 to 1.5 μm. Therefore, quantification of axon damage included only those YFP-filled swellings >2.5 μm in width with peak fluorescent value (measured as maximum pixel intensity) of at least 25% greater than adjacent axons, which was above the 95% confidence interval of the YFP fluorescence intensity of adjacent normal-appearing axons. Cell bodies were distinguished using 4′,6-diamidino-2-phenylindole (DAPI) nuclear staining. Axonal varicosities included YFP-filled swellings along an axon and terminal end bulbs, that is, swellings at the end of an axon segment. Analysis included six to eight sections per mouse.

### *Thy1-YFP-16* mice: analysis of neuroinflammation

Mice perfused at 3 days following final rBlast or rSham procedures were further analyzed using immunohistochemistry in coronal sections at levels between +0.5 and −0.5 mm relative to bregma. The CC ROI extended from the midline bilaterally to under the peak of the cingulum.^21^ Astrocytes were evaluated by immunostaining for glial fibrillary acidic protein (GFAP; polyclonal rabbit anti-GFAP; 1:1000; DAKO, Carpinteria, CA, USA; Agilent Cat# N1506, RRID:AB_10013482). Microglia/macrophages were identified using polyclonal rabbit antibody against ionized calcium binding adaptor molecule 1(IBA1; 1:1000; Wako, Richmond, VA, USA; Cat# 019-19741, RRID:AB_839504). All tissue sections were counterstained with DAPI nuclear stain (Sigma-Aldrich., St. Louis, MO, USA; D9542). For quantification, images within the CC ROI were acquired with a 10 × objective on an Olympus IX-70 microscope using a SPOT RT3 camera. ImageJ software was used to threshold fluorescence levels to quantify the area of immunolabeling above background.^28^ Images were also acquired with a 40 × objective to examine the morphology of IBA1 immunolabeled cells to compare the density of resting or activated cells.^20,35,37^ Analysis included six to eight sections per mouse.

### *NG2CreER;mTmG* mice: analysis of myelin

Cohorts of *NG2CreER;mTmG* littermates received rBlast or rSham procedures. TMX (10 mg in 20 mg/mL corn oil suspension; Millipore, Sigma-Aldrich; T5648) was administered via oral gavage on day 2 and 3 after the final blast or sham procedure.^21,38^ TMX induces *Cre recombinase* mediated deletion of a stop codon switches transcription from constitutive expression of membrane-localized tdTomato (mT) to NG2 driven expression of membrane-localized GFP (mG). Fluorescence was quantified in coronal sections at levels between +0.5 and −0.5 mm relative to bregma. The CC ROI extended from the midline bilaterally to under the peak of the cingulum.^21^ Images were acquired on an Olympus IX-70 microscope using a SPOT RT3 camera. ImageJ software was used to threshold the signal to above background and quantify the area of membrane fluorescence from the CC ROI, as previously detailed for CC mG labeling and for immunolabeling of myelin.^21,28^ Confocal images were acquired with a 40 × objective to examine the morphology of membranes labeled with mG and mT. Additional tissue sections were immunostained for total myelin based on myelin oligodendrocyte glycoprotein (MOG; mouse monoclonal, 1:100; EMD Millipore, Burlington, MA, USA; mab5680; RRID:AB_1587278).

### Statistical analysis

Mice were allocated to the rBlast or rSham procedures using the random number generator function in Microsoft Excel. Investigators were blinded to animal injury condition during tissue analysis until after completion of the data analysis. GraphPad Prism 8.0 software (RRID:SCR_002798) was used for statistical analysis and graphing. Bar graphs demonstrate means with standard error of the means (SEMs) as well as symbols for individual mouse values. Two-way ANOVA with repeated measures, for CC and cingulum within same subjects, and Sidak's post hoc multiple comparisons test were used for axonal varicosity comparisons. An unpaired Student's *t* test was used to compare immunolabeling of astrocytes and microglia between groups. Two-way ANOVA with Sidak's post hoc multiple comparisons test was used at each time-point for comparing mG fluorescence and CC width between rSham and rBlast in *NG2CreER;mTmG* mice. Statistical significance was set at values of *p* < 0.05.

## Results

### Screening in *Thy1-YFP-16* fluorescent reporter mice detects damaged axons following repetitive blast exposure

**FIG. 1.**
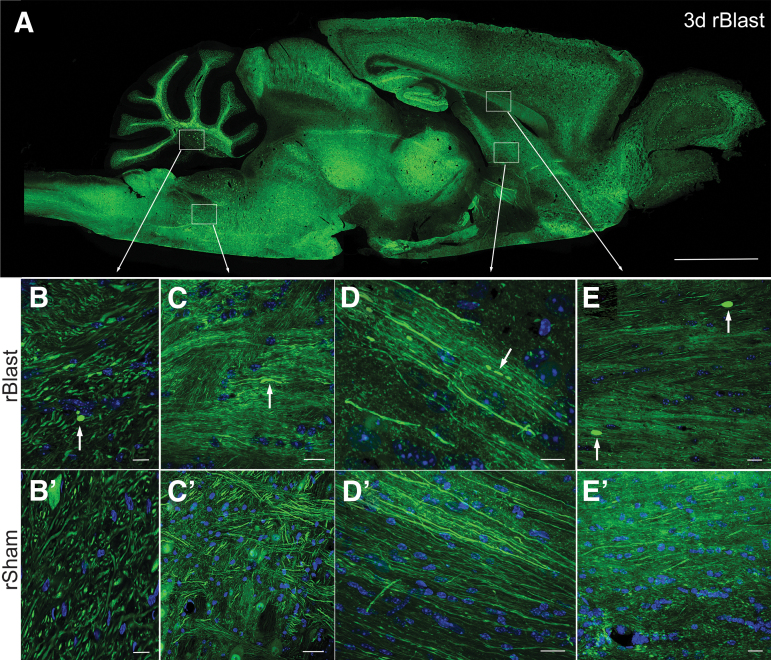
Broad, sensitive screening for axon damage in brain sections after repetitive blast exposure using *Thy1-YFP-16* mice. **(A)** Sagittal section from a *Thy1-YFP-16* mouse at 3 days after rBlast exposure shows widely distributed neuron cell bodies and processes expressing yellow fluorescent protein (YFP; shown in green). **(B–D’)** Sagittal high-magnification confocal microscope images of axons in white matter tracts in distributed brain regions of rBlast mice (B–D) and matching regions in rSham mice (B’–D’). YFP shown in green. Nuclear stain 4′,6-diamidino-2-phenylindole (DAPI) shown in blue. (B) Example of YFP accumulated in damaged axon to illustrate a terminal end bulb (arrow; transverse view) in the cerebellum of an rBlast mouse. Note that other YFP-filled oval and elongated structures are axons shown in angled cuts in both rBlast (B) and rSham (B’) mice. The intense YFP of damaged axons is distinct from larger, more diffuse labeling in neuron cell bodies (adjacent to B’ label). Examples from rBlast mice of YFP in an axonal thickening (arrow; longitudinal view) in the pons region of the brainstem (C) and in fragmented axons (arrows; longitudinal view) in the column of the fornix (D). **(E,E’)** Coronal confocal image showing corpus callosum axons in longitudinal view with two examples of intense YFP labeling in large terminal end bulbs in an rBlast section (E; arrows), in comparison with an rSham section (E’). Low-power image (A) is representative of images from three *Thy1-YFP-16* mice examined in full sagittal sections. Higher-power images (B–E’) were acquired from sections of these or additional *Thy1-YFP-16* mice examined in coronal sections for quantification in Figure 2. Scale bars = 2 mm (A), 20 μm (B–E and B’–E’).

The effects of repetitive blast exposures on axons were examined in *Thy1-YFP-16* fluorescent reporter mice that express YFP in diverse neuronal populations but in only a subset of those neurons, which allows visualization of individual axons (Fig. 1). Confocal microscopy identified YFP-filled axonal swellings, which can detect axonal injury ranging from early reversible damage through terminal axon disconnection.^22,23^ As an initial qualitative assessment, tissues were screened in parasagittal sections of the brain and brainstem at 3 days after the last of five repetitive blast exposures (rBlast; Fig. 1A–E) that were delivered at 24-h intervals, or after matched procedures (rSham; Fig. 1B’–E’) without deploying the blast. YFP-filled axonal swellings were observed in rBlast mice in diverse brain regions, including the cerebellum (Fig. 1B), pons (Fig. 1C), fornix (Fig. 1D), and CC (Fig. 1E). Overall, axons with YFP-filled swellings were found diffusely distributed in the brain of rBlast mice.

### Repetitive blast exposure induces acute axon damage in white matter tracts

**FIG. 2.**
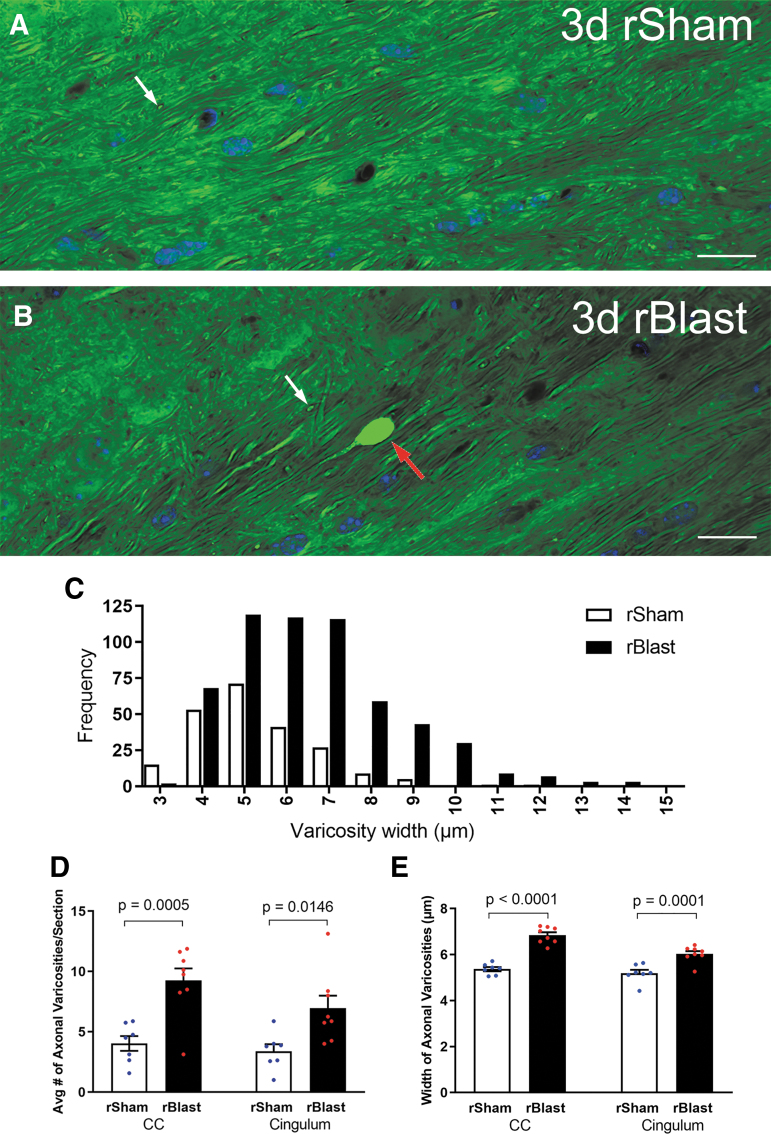
Repetitive blast exposure increases axon damage in the corpus callosum (CC) and adjacent cingulum. **(A,B)** Confocal images of coronal sections of the CC from *Thy1-YFP-16* mice at 3 days after repetitive sham procedures (rSham; A) or repetitive blast exposure (rBlast; B) showing high magnification of yellow fluorescent protein signal (YFP) within axons and showing 4′,6-diamidino-2-phenylindole (DAPI) nuclear stain in blue. The rSham mice exhibit relatively uniform YFP signal filling of axons viewed longitudinally (A). There are small variations in diameter along the length of the axons. In addition, YFP signal is visible in axons of crossing fibers cut transversely (A; white arrow). An example in an rBlast mouse of an axon with a large, intensely fluorescent YFP-filled enlargement indicative of axon damage (B; red arrow) that is distinct from crossing fiber axons (B; white arrow). **(C)** Analysis of the frequency distribution indicates an increase in both the number and diameter of YFP accumulations in the CC and cingulum of rBlast mice compared with rSham controls. (**D)** The rBlast significantly increases the incidence of damaged axons with abnormal YFP localization (thickenings, varicosities, and terminal end bulbs) in the CC and cingulum, compared with rSham mice. **(E)** YFP accumulations in axonal varicosities are significantly larger in rBlast compared with rSham mice (B). Values are mean ± standard error of the mean (SEM). Two-way analysis of variance (ANOVA) was used with repeated measures, for CC and cingulum within same subjects, and Sidak's post hoc multiple comparisons test. For the procedures, mouse numbers were: rSham group, *n* = 7 and rBlast group, *n* = 8. Scale bars = 20 μm (A and B).

Further analysis of blast exposure in *Thy1-YFP-16* mice focused on the CC and overlying cingulum, which had readily detected YFP-filled axonal varicosities in the rBlast mice (Fig. 1) and are clinically relevant areas of damage identified in human blast patients.^10,17^ These long white matter tracts also run perpendicular to one another, which is of interest relative to screening for broad effects of blast waves across brain regions. YFP-filled axonal varicosities were visualized at high resolution using confocal microscopy of coronal tissue sections through the CC and cingulum at 3 days after the final rBlast or rSham procedure (Fig. 2). The rSham mice showed YFP signal distributed within axons running longitudinally in the coronal plane or within interspersed crossing fibers (Fig. 2A). The rBlast mice exhibited distributed YFP signal in most axons but also rare axons with large accumulations of YFP (Fig. 2B). 3D reconstruction of confocal image stacks identified distinctly large axonal swellings to be terminal end bulbs. YFP accumulations were more frequent and often larger in rBlast mice relative to rSham mice (Fig. 2C–E).

### Repetitive blast exposure induces neuroinflammation in the corpus callosum

**FIG. 3.**
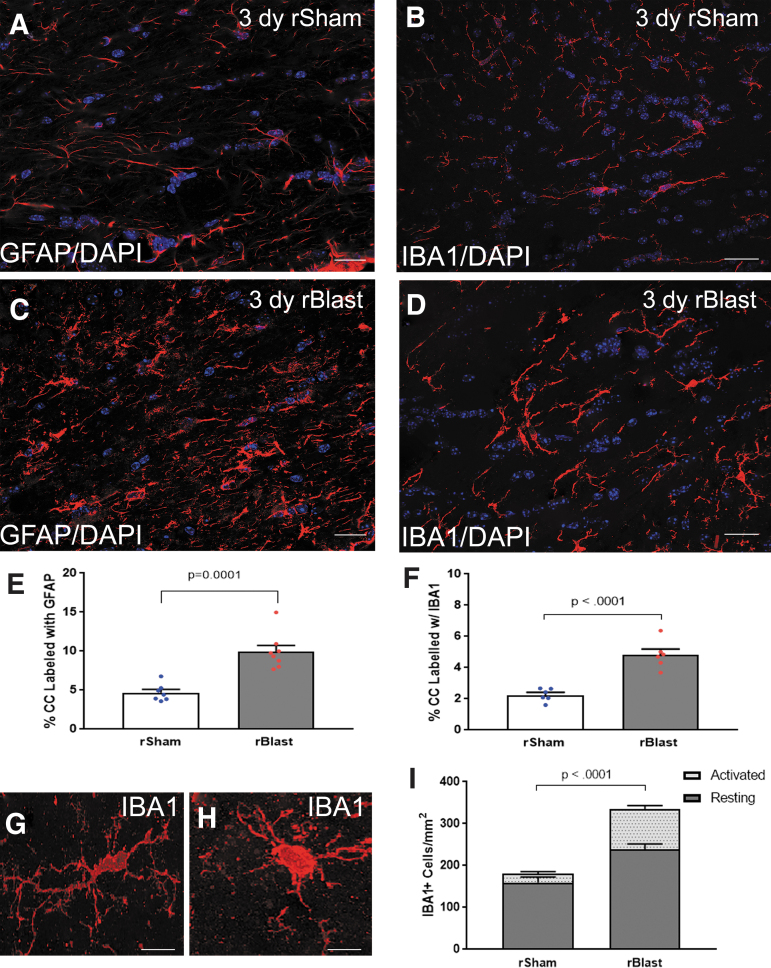
Neuroinflammation is significantly increased in the corpus callosum (CC) after blast exposure. **(A–D)** Immunohistochemistry to detect glial fibrillary acidic protein (GFAP) in astrocytes and ionized calcium binding adaptor molecule 1 (IBA1) as a marker of microglia/macrophages. Confocal microscope images of coronal CC sections from *Thy1-YFP-16* mice at 3 days after repetitive sham procedures (rSham; A,B) or repetitive blast exposure (rBlast; C,D). Astrocytes (pseudocolored white) and microglia/macrophages (red) have thicker processes with more intense immunoreactivity in rBlast mice as compared with rShams. Nuclear stain 4′,6-diamidino-2-phenylindole (DAPI) shown in blue. **(E,F)** The rBlast exposure significantly increased immunoreactivity for GFAP (E) and IBA1 (F). **(G,H)** Representative confocal images showing IBA1+ cells as examples of resting state morphology with an elongated cell body and numerous processes (G) or activated morphology with a more rounded and intensely immunolabeled cell body and processes (H). **(I)** rBlast exposure significantly increases the number of IBA1+ cells in the CC. Values are mean ± standard error of the mean (SEM). Student's *t* test was used to compare between groups. GFAP groups include rSham, *n* = 7; rBlast, *n* = 8. IBA1 groups include rSham, *n* = 6; rBlast, *n* = 7. Scale bars = 20 μm (A–D) and 5 μm (G,H).

To determine whether a neuroinflammatory response accompanied blast-induced axon pathology, coronal sections were immunolabeled for GFAP as a marker for astrocytes and IBA1 for microglia/macrophages in *Thy1-YFP-16* mice at 3 days following final rBlast or rSham procedure (Fig. 3). The astrocytes and microglia have fine processes in the CC of rSham mice (Fig. 3A,B). Following rBlast exposure, reactive astrocytes and activated microglia exhibited shorter, thicker processes with increased immunoreactivity, indicative of a mild innate immune response (Fig. 3C,D). Quantitative analysis demonstrated a significant increase of immunoreactivity for GFAP and IBA1 in rBlast mice when compared with rSham (Fig. 3E,F). Further, rBlast exposure increased the density of IBA1 immunolabeled cells (Fig. 3G–I). These results provide evidence that rBlast exposures produce an acute neuroinflammatory response in the same tissue regions that exhibit axon damage.

### Screening new myelin formation in *NG2CreER;mTmG* reporter mice following repetitive blast exposure

**FIG. 4.**
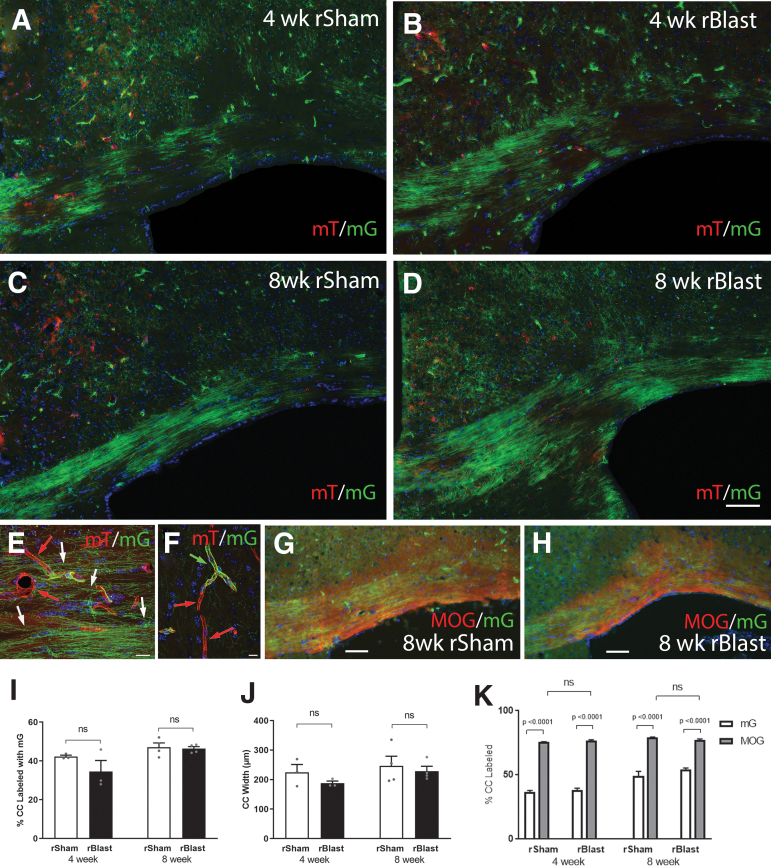
Myelin membrane synthesis in the corpus callosum (CC) after repetitive blast exposure in *NG2CreER;mTmG* reporter mice. *NG2CreER;mTmG* mice were given tamoxifen (TMX) on days 2–3 after repetitive blast (rBlast) or sham (rSham) procedures. TMX induces recombination to stop expression of membrane-localized tdTomato (mT) and initiate expression of membrane-localized green fluorescent protein (mG) driven from the NG2 promoter (NG2mG). NG2 expression drives genetic mG fate-labeling of oligodendrocyte progenitors that is maintained in newly generated oligodendrocytes and their myelin membranes. **(A–D)** Representative images of mG (green) labeled membranes in coronal sections through the CC at 4 weeks (A,B) or 8 weeks (C,D) after rSham (A,C) or rBlast (B,D). (**E,F)** High-magnification confocal microscope images within the CC (E) and cortex (F). Constitutive mT (red) labeling is visible in membranes of non-recombined cells, including blood vessels (E; red arrows). NG2 fate-labeled oligodendrocytes extend myelin-like membranes (E; white arrows) along axons. NG2 fate-labeled pericyte (F; green arrow) extend processes around blood vessels (F; red arrows). Nuclear stain 4′,6-diamidino-2-phenylindole (DAPI) shown in blue. (**G,H)** Representative images of mG fluorescence (green) in newly synthesized myelin membranes along with immunolabeling for myelin oligodendrocyte glycoprotein (MOG) in new and persisting myelin membranes (red). The mG membranes represents only a subset of the total myelinated area of the CC. **(I)** rBlast did not significantly alter the proportion of mG-labeled myelin membranes synthesized in the CC at either time point. **(J)** rBlast exposure did not result in significant CC atrophy at either time-point. **(K)** Quantification of mG labeling relative to total MOG immunolabeled myelin in the same sections. The myelinated fraction in the CC is significantly greater than the mG new myelin and is not changed by rBlast exposure. Values are mean ± standard error of the mean (SEM). Two-way analysis of variance (ANOVA) with Sidak's post hoc multiple comparisons test. Four-week groups include rSham, *n* = 3; rBlast, *n* = 3. Eight-week groups include rSham, *n* = 4; rBlast, *n* = 4. Scale bars = 100 μm (A–D, shown in D), 20μm (E–H).

In addition to axon damage and neuroinflammation, white matter microstructural effects of blast detected by MRI could also involve myelin membranes that ensheath axons.^14,39,40^ We previously showed that *NG2CreER;mTmG* myelin reporter mice revealed an increase of new myelin synthesis in the CC over a 4-week period after single impact TBI, possibly indicating replacement of damaged myelin sheaths.^21^ Therefore, *NG2CreER;mTmG* mice were used to quantify new myelin synthesis following rBlast exposure (Fig. 4). TMX was administered on days 2 and 3 after the final blast or sham procedure, which corresponds with the time-point for analysis of axon damage (Figs. 1–3).

Fluorescent green mG labeling identified new membranes synthesized after TMX induction. Clusters of green myelin membranes were distributed in the CC at 4 weeks after the final rBlast/rSham procedure (Fig. 4A,B) with further myelin formation by 8 weeks (Fig. 4C,D). These clusters are as expected with genetic fate-labeling of NG2 expressing progenitors that generate new oligodendrocytes, which each form myelin sheaths around many axons. Higher magnification showed green myelin aligned along axons (Fig. 4E). Green membranes extending along blood vessels are also occasionally observed due to NG2 expression in pericytes (Fig. 4F). Immunolabeling for MOG to detect myelin shows the presence of persisting myelin as well as newly synthesized mG-labeled membranes (Fig. 4G,H). Quantification of mG membrane labeling in rSham mice showed substantial formation of new myelin membranes continues in adulthood (Fig. 4I). This findings is in agreement with studies that show CC myelination continues well beyond 16 weeks of age,^36^ which is the age match of 8 weeks post-blast. rBlast exposure did not change the extent of new myelin membrane synthesis in the CC after either 4 or 8 weeks (Fig. 4I). Further, in our prior study using *NG2CreER;mTmG* mice, single-impact TBI resulted in reduced CC width at 8 weeks,^21^ which was not found after rBlast exposure (Fig. 4J). Finally, rBlast exposures does not produce significant loss of CC myelin, as detected by MOG immunolabeling (Fig. 4K).

## Discussion

The ability to detect subtle and distributed pathological features is vital to determining the effects of blast exposure on the brain. This study utilized two distinct fluorescent reporter mouse lines to assess components of potential axon and myelin white matter injury after rBlast exposures. The results demonstrate that rBlast exposure produces acute axon damage in multiple white matter tracts of the brain, as detected by screening in *Thy1-YFP-16* mice. Acute axon damage was accompanied by an innate immune response of mildly reactive astrocytes and microglia. However, this white matter pathology did not lead to later effects on myelin synthesis, an indicator of myelin repair and remodeling, which was tracked *in vivo* after blast exposure using *NG2CreER;mTmG* mice.

To model the blast wave component of low-level exposures to explosives or heavy artillery, the current experiments used an ABS device to generate controlled high-fidelity simulation of both positive and negative phases of “free-field” blast.^33,41–44^ Studies using single-blast exposures in the ABS in adult rats have produced long-term behavioral effects.^44^ Similar ABS exposures can alter the cerebral vasculature and the blood–brain barrier.^41,42^ Blast exposure can also alter cellular pathology including expression of markers for gliosis and myelinated axons (CNPase and neurofilament H), but appears to vary with the degree of head immobilization.^43^ Indeed, single ABS exposure at 15 psi using a mesh to position the mouse in the prone position and minimize head movement resulted in a low-level blast frontal exposure that did not induce detectable microglial activation in the CC.^33^ The current studies used this same mesh procedure to similarly secure and position the mouse in the ABS. Our prior studies of concussive mild TBI in mice demonstrated persistent neuroinflammation in the CC and adjacent cortex after one impact per day on 5 consecutive days.^20^ With repetitive exposure pattern and increasing the ABS exposure to approximately 20 psi, we find a significant but low-level neuroinflammatory response in both astrocytes and microglia (Fig. 3). This CC neuroinflammation indicates a repetitive blast effect that warrants investigation of axon and myelin pathology.

*Thy1-YFP-16* mice facilitated detection of axonal varicosities, including terminal end bulbs, which are significantly increased at 3 days after the final ABS exposure in the CC and cingulum (Figs. 1 and 2). Previous studies in rodent models of blast exposure found electrophysiological deficits and axon damage within white matter tracts along with behavioral deficits.^45,46^ Axon damage after blast exposure appears to be subtle and not readily detected by immunohistochemistry.^47–49^ The current YFP approach revealed individual damaged axons in mice even when exposed to a relatively low mean peak incident overpressure of approximately 20 psi (Fig. 2).

Our findings of acute neuroinflammation and axon damage in the CC led us to further examine myelination as an indicator of longer-term white matter injury after rBlast that is consistent with neuroimaging DTI findings in patients who have been experienced blasts. Myelin has been shown to be disrupted in TBI and myelin repair may have profound implications for recovery of axon function.^50^ Studies with open field explosive blasts reported CC pathology with increased silver staining of damaged axons and ultrastructural evidence of myelin disruption.^40^ However, this electron microscopic technique to evaluate myelin can only sample small regions. Additionally, the ability to monitor the dynamic turnover of myelin membranes is important to capture in evaluating potential myelin repair. In the current study, the use of *NG2CreER;mTmG* mice enabled quantification of *in vivo* myelin synthesis during specific intervals after blast exposures. During these periods, rBlast exposure did not produce late-phase effects on synthesis of new myelin membranes or induce CC atrophy (Fig. 4 G,H). This result is in agreement with an overall mild level of white matter injury based on the limited degree of neuroinflammation and rare axon damage in the CC of rBlast mice. Possibly, TMX on days 2 and 3 could lessen blast pathology at 8 weeks. However, at 8 weeks after a concussive TBI, reduced CC width was found in two separate mouse studies in which one did not involve TMX and the other administered TMX using the current protocol.^21,51^

The advantages of mouse models to leverage the genetic labeling of cellular components are balanced by the limitations of small size and lack of complexity of the mouse brain. Mouse models are most useful as tools to screen for pathology that may be otherwise hard to distinguish. These findings in mice should facilitate further development of techniques applicable to higher mammals and humans, and interpretation of clinically applicable techniques such as white matter microstructure effects identified by MRI DTI. The devices available to model blast exposures increase reproducibility by reducing experimental variables and so do not fully replicate the human experience. In the broadest sense, the blast field overall is limited by the need for further studies to more fully establish the human neuropathology of TBI associated with blast exposure.

## Conclusion

Evaluating effects of low-level repetitive blast exposure is challenging due to potentially subtle and diffuse pathology involving multiple cell types and tissues. *Thy1-YFP-16* mice detected axon damage in white matter tracts across multiple brain regions. *NG2CreER;mTmG* mice detected new myelin synthesis and provided the ability to visualize the neurovascular-pericyte unit. Taken together, this study demonstrates that fluorescent reporter mice are effective tools for evaluating cellular pathology of blast-induced neurotrauma and that repetitive low-level blast exposures produce infrequent but significant axon damage along with neuroinflammation in white matter.

## Abbreviations Used

3D: three-dimensional
ABS: Advanced Blast Simulator
ANOVA: analysis of variance
CC: corpus callosum
DAPI: 4′,6-diamidino-2-phenylindole
DTI: diffusion tensor imaging
GFAP: glial fibrillary acidic protein
GFP: green fluorescent protein
IBA1: ionized calcium binding adaptor molecule 1
mG: membrane-localized GFP
MOG: myelin oligodendrocyte glycoprotein
MRI: magnetic resonance imaging
mT: tdTomato
OPC: oligodendrocyte progenitor cell
ROI: region of interest
SEM: standard error of the mean
TBI: traumatic brain injury
TMX: tamoxifen
YFP: yellow fluorescent protein
β-APP: β-amyloid precursor protein
